# Assessing the Color Stability of Polymethyl Methacrylate (PMMA) Dental Restorations Polished With a Novel Polishing Agent Derived From Pulverized Old Alginate Impressions

**DOI:** 10.7759/cureus.67614

**Published:** 2024-08-23

**Authors:** Rahul Koppaka, Nabeel Ahmed, Urvi R Echhpal

**Affiliations:** 1 Department of Prosthodontics and Implantology, Saveetha Dental College and Hospitals, Saveetha Institute of Medical and Technical Sciences, Saveetha University, Chennai, IND

**Keywords:** polymethyl methacrylate (pmma), pmma, polishing, pumice, algishine, acrylic resin

## Abstract

Introduction: Interim restorations are essential in restorative dentistry, serving as temporary solutions until permanent restorations can be placed. Polymethyl methacrylate (PMMA) is a promising solution for customizing teeth for removable dentures to match the exact requirements of patients. The color stability of these restorations is critical for patient satisfaction. PMMA is a widely used material for interim restorations due to its favorable properties. The study compares the color stability of PMMA interim restorations polished using traditional pumice versus Algishine, a novel polishing agent derived from pulverized old alginate impressions.

Materials and methods: The 3-D design software Geomagic Design X (3D Systems, Rock Hill, CA) created a standard tessellation language file of 2-cm radius circles. Sixty PMMA samples were milled and divided into two groups of 30 each. Group A samples were polished using pumice, while group B samples were polished with Algishine. Baseline color measurements were taken using a spectrophotometer (VITA Easyshade V, VITA Zahnfabrik, Bad Säckingen, Germany). The samples were then subjected to staining with coffee, tea, and red wine solutions for 30 days, simulating oral conditions. Post-staining color measurements were taken, and color changes (ΔE) were calculated at the seven-day (t1) and one-month (t2) mark. The Shapiro-Wilk test assessed normality, followed by a two-way ANOVA test to compare color change values at different time points.

Results: At t1 (seven days), there were no significant differences between groups A and B in the coffee and tea staining groups. However, significant differences were observed in red wine staining, with group B exhibiting lower ΔE values (0.14 ± 0.067) compared to group A (0.38 ± 0.076) (p < 0.01).

At t2 (30 days), significant differences were noted in all staining groups. Group B consistently showed lower ΔE values: coffee (0.125 ± 0.084 vs. 0.236 ± 0.015, p < 0.01), tea (0.254 ± 0.087 vs. 0.391 ± 0.015, p < 0.01), and red wine (1.174 ± 0.045 vs. 1.309 ± 0.074, p < 0.01), indicating superior resistance to staining compared to group A.

Discussion: The results suggest that Algishine is more effective than pumice in maintaining the color stability of PMMA interim restorations. The novel polishing agent derived from old alginate impressions enhances esthetic longevity and provides an eco-friendly solution for recycling dental material waste.

Conclusion: Algishine performs superiorly in preserving the color of PMMA interim restorations against common staining agents. Its application can potentially improve patient satisfaction and contribute to sustainable dental practices.

## Introduction

In today's world, dentists and patients have a variety of options when it comes to choosing interim restorations. Several factors, like esthetic and functional requirements, determine the material of choice. Commonly used materials include self-cure acrylic, computer-aided design/computer-aided manufacturing polymethyl methacrylate (PMMA), and composite resin materials [1].

Interim dental restorations protect the prepared tooth, restore function and esthetics, and maintain space for the final restoration [2]. PMMA is the material of choice for many practitioners due to its favorable physical properties and ease of fabrication [3]. However, one of the challenges with PMMA is its susceptibility to discoloration over time, particularly when exposed to various staining agents in the oral environment [4]. Polishing is crucial in minimizing discoloration, as a smoother surface is less prone to stain accumulation [5].

Traditionally, pumice has been widely used to polish acrylic resins. This volcanic ash-derived powder effectively smooths rough surfaces, achieving a polished finish. However, its use poses ecological challenges, including habitat disruption and carbon emissions from mining. Moreover, being a finite resource, ongoing extraction raises sustainability concerns. In response to these environmental issues, there is growing interest in sustainable alternatives [6].

This study introduces Algishine, a new polishing agent made from recycled pulverized alginate impressions. Alginate, sourced from seaweed, is commonly used in dental practices to make teeth and oral cavity impressions. It primarily comprises diatomaceous earth particles that act as an abrasive agent. Algishine transforms this waste into a polishing material, typically discarded after use, promoting environmental sustainability and resource efficiency.

This study aims to evaluate the effectiveness of Algishine compared to pumice in maintaining the color stability of PMMA interim restorations. The null hypothesis states that there is no significant difference in the color stability of PMMA polished using pumice and Algishine.

## Materials and methods

Sample size calculation

This study involved an in vitro experimental design. The sample size was determined using the G*Power software version 3.1.2 from Heinrich Heine University, Düsseldorf, Germany, setting an alpha level of 0.05 and a test power of 0.80. Consequently, the sample size was 60, which was then divided into two groups of 30 per group. These 30 samples were then subdivided into three groups of 10 each for staining with tea, coffee, and red wine, respectively.

Preparation of Algishine

Alginate impressions were disinfected with 2% glutaraldehyde for 30 minutes. After disinfection, the alginate impressions were collected and ground into a fine powder. The grinding was done using a Foshan Xingle Machinery XL-88 device with a sealable chamber and a freely rotating blade inside. This process resulted in the production of a dehydrated powder [7].

Preparation of samples

**Figure 1:**
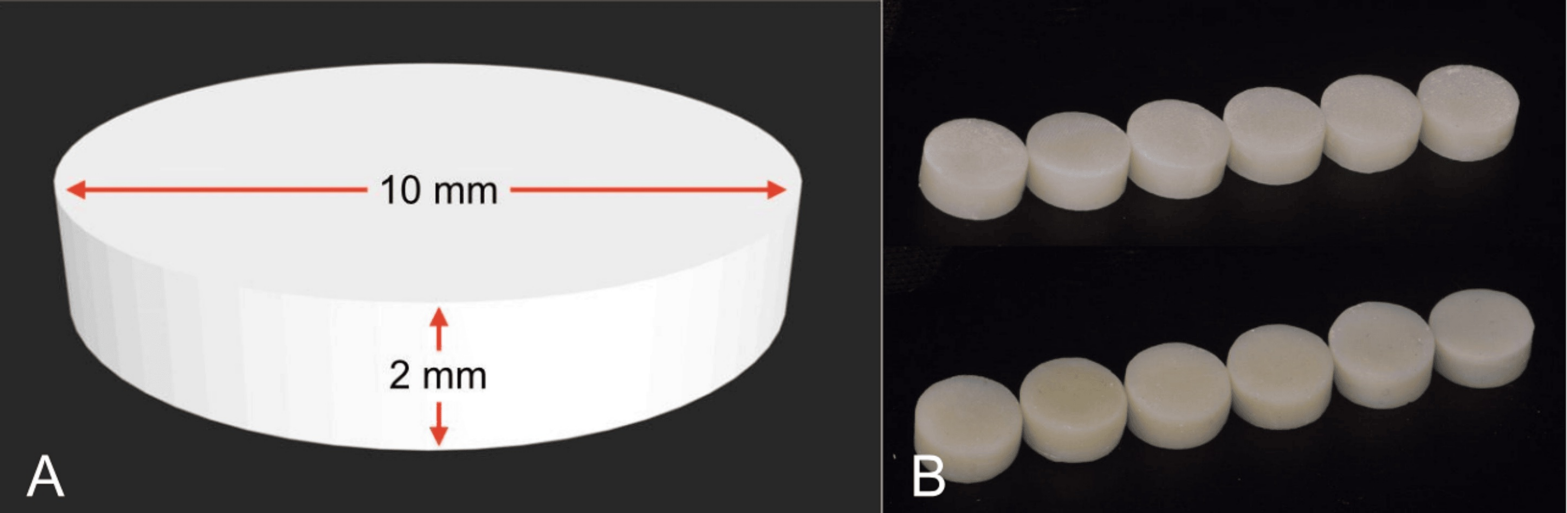
(A) STL file of the sample. (B) Milled specimen. The STL sample was designed to be 10 mm in diameter and 2 mm in width. This digital design was then milled in PMMA material STL: standard tessellation language; PMMA: polymethyl methacrylate

Sixty samples in the shape of discs (10 mm in diameter and 2 mm thick) were prepared using the 3-D design software Geomagic Design X (3D Systems, Rock Hill, CA). The standard tessellation language files were saved and then nested using the imes icore software, following which they were milled in PMMA using a 5-axis milling machine (imes icore 350i, Hessen, Germany). The samples were divided into two groups of 30 each: group A and group B (Figure 1).

Polishing protocol

Samples were polished with sandpaper of various grits, 100→400→1,000. Shofu Acrylic Polishing bur kit (Shofu, Kyoto, Japan) was used with four burs: extra coarse (gray), coarse (blue), fine (dark green), and extra fine (light green). Group A samples were polished using a low-speed pumice paste applied with a rotary brush. Group B samples were polished with Algishine. The same polishing technique and duration were applied to both groups to ensure consistency. The samples were then polished with a buff (wet buffing was followed by dry buffing).

Baseline color measurement

All samples were measured for baseline color using a spectrophotometer (VITA Easyshade V, VITA Zahnfabrik, Bad Säckingen, Germany) under standardized lighting conditions. The CIE Lab* color space system was used to record the values, where L* indicates lightness, a* represents the red-green coordinate, and b* represents the yellow-blue coordinate.

Staining protocol

Testing for color stability in this study included coffee, tea, and red wine solutions. The tea, coffee, and red wine solutions were prepared according to the following protocols: Following the manufacturer's guidelines, 8 g of coffee was combined with 400 mL of boiling distilled water to prepare the coffee solution [8]. This was based on the standard value of approximately 3.2 cups, equivalent to 15-20 minutes per cup. For the tea solution, two tea bags were steeped in 400 mL of boiling water and allowed to cool for 15 minutes, after which they were filtered using gauze. Sette by Fratelli Wines was used to prepare the red wine solution.

These containers were housed in an incubator set at 37 ± 1°C. The solutions were changed and refreshed every three days to maintain concentration and stirred daily to prevent particle settling. A single operator prepared all solutions to ensure consistency and minimize methodological variability.

Post-staining color measurement

Following the staining period, the samples were rinsed with distilled water and dried using absorbent paper. Post-staining color measurements were conducted using the same spectrophotometer and under the same conditions as the baseline measurements.

Calculation of color change (ΔE)

The color alteration (ΔE) was evaluated with a spectrophotometer (SpectraMagic NX, RM2002QC, Konica Minolta Corp., Ramsey, Japan) with a white background. Assessments were conducted at two separate time points: after one week (t1) and after one month (t2) of immersion, with the subsequent analysis being standardized by using a positioning index made using autopolymerizing resin. The ΔE value was computed using the formula:

ΔE = √(ΔL)^2 ^+ (Δa)^2 ^+ (Δb)^2^​

Statistical analysis

The Shapiro-Wilk test was employed to ascertain whether the data adhered to a normal distribution. A two-way ANOVA test was performed to compare color stability changes across groups at various time points.

## Results

At t1, when immersed in coffee, the mean ΔE value for group A was 0.025 ± 0.055, while group B showed a mean ΔE of 0.024 ± 0.058. The difference was not statistically significant (p > 0.01). When immersed in tea, group A exhibited a mean ΔE value of 0.109 ± 0.032, compared to group B's mean ΔE of 0.092 ± 0.044. This difference was not statistically significant (p > 0.01). When in red wine, group A had a mean ΔE of 0.38 ± 0.076, whereas group B demonstrated a mean ΔE of 0.14 ± 0.067. The statistical analysis confirmed the significance of this difference (p < 0.01).

At t2, coffee staining demonstrated the mean ΔE value for group A as 0.236 ± 0.015, while group B showed a mean ΔE of 0.125 ± 0.084. The difference was statistically significant (p < 0.01). In the tea, group A exhibited a mean ΔE value of 0.391 ± 0.015, compared to group B's mean ΔE of 0.254 ± 0.087. This difference was also statistically significant (p < 0.01). For red wine staining, group A had a mean ΔE of 1.309 ± 0.074, whereas group B demonstrated a mean ΔE of 1.174 ± 0.045. The statistical analysis confirmed the significance of this difference (p < 0.01) (Table 1).

**Table 1:** Mean and standard deviation of ΔE within groups A and B at t1 (seven days) and t2 (one month)

Groups	Timing	Staining agent	N	Mean	Standard deviation
Group A (pumice)	t1	Coffee	10	0.025	0.055
		Tea	10	0.109	0.032
		Red wine	10	0.38	0.076
	t2	Coffee	10	0.236	0.015
		Tea	10	0.391	0.015
		Red wine	10	1.309	0.074
Group B (Algishine)	t1	Coffee	10	0.024	0.058
		Tea	10	0.092	0.044
		Red wine	10	0.14	0.067
	t2	Coffee	10	0.125	0.084
		Tea	10	0.254	0.087
		Red wine	10	1.174	0.045

Between t1 and t2, data were analyzed to check each solution's staining rate. At the initial time point (t1), red wine showed a statistically significant effect on color stability (p = 0.000), whereas coffee (p = 0.342) and tea (p = 0.083) did not. However, at the subsequent time point (t2), all three staining agents, coffee (p = 0.001), tea (p = 0.000), and red wine (p = 0.000), exhibited statistically significant effects on color stability. This indicates that the staining impact of these liquids becomes more pronounced over time (Table 2).

**Table 2:** Standard error and significance within both group A and group B at t1 (seven days) and t2 (one month) Two-way ANOVA was used to perform the statistical tests Group A: samples polished with pumice; group B: samples polished with Algishine

Timing	Staining liquid	Standard error	Significance
t1	Coffee	0.0057	0.342
	Tea	0.1056	0.083
	Red wine	0.0202	0.000
t2	Coffee	0.02	0.001
	Tea	0.0183	0.000
	Red wine	0.0168	0.000

This study revealed no significant difference between the coffee and tea groups on the seventh day. In comparison, significant differences in color stability between the two polishing agents were seen on the seventh day in the red wine group and all three groups on day 30. Group B (Algishine) samples consistently showed lower ΔE values, indicating better resistance to staining compared to group A (pumice).

## Discussion

The main objective of this in vitro analysis was to evaluate the color stability of PMMA interim restorations polished using two different agents: traditional pumice and a novel agent, Algishine, derived from pulverized old alginate impressions. The findings demonstrate that Algishine significantly outperforms pumice in maintaining the color stability of PMMA restorations when exposed to common solutions like coffee, tea, and red wine after 30 days. Therefore, the null hypothesis was rejected.

The superior performance of Algishine can be attributed to several factors. Algishine's finer particle size than pumice allows for a smoother finish on the PMMA surface, minimizing the adherence of staining agents and reducing the likelihood of discoloration [9]. Additionally, the polishing process with Algishine may produce a uniform and less porous surface, which is critical in preventing stain penetration. The chemical properties of alginate could also play a role in enhancing the surface characteristics of PMMA. Alginate, a naturally occurring biopolymer, has inherent hydrophilic properties, possibly contributing to a less stain-retentive surface [10]. When pulverized and used as a polishing agent, these properties could translate into improved resistance to common staining agents.

Moreover, Algishine offers a sustainable alternative to traditional polishing methods by recycling old alginate impressions. This reduces waste and aligns with environmentally conscious practices in dental offices. Utilizing waste materials from alginate impressions addresses environmental concerns and provides a cost-effective resource for dental polishing.

The findings from this study have significant implications for dental practice. Interim restorations are expected to maintain their esthetic appearance for the duration of their use. Patients often have high esthetic expectations, even for temporary solutions. Using Algishine, dental practitioners can ensure that interim restorations retain their color and appearance, enhancing patient satisfaction. Patients are more inclined to follow treatment plans when pleased with the esthetic results. By minimizing discoloration, Algishine can improve the overall patient experience and compliance with interim restorations, particularly in long-term provisional scenarios.

While the initial investment in developing Algishine from recycled materials might be higher, the long-term cost benefits are substantial. Dental practices can reduce expenditures on polishing agents by reusing materials that would otherwise be discarded. These cost savings can be passed on to patients or reinvested into the practice for other improvements.

To understand why Algishine performs better, it is essential to consider the polishing mechanism. The surface roughness of PMMA plays a critical role in stain retention [11]. Polishing with Algishine results in a significant reduction in surface roughness compared to pumice. The interaction between the polished PMMA surface and staining agents is crucial [12]. Algishine-polished surfaces exhibit lower adsorption of staining molecules due to reduced roughness and different surface chemistry. This hypothesis could be tested by analyzing the surface energy and wettability of PMMA samples polished with both agents [13]. Additionally, the durability of the polished surface under oral conditions is another factor. Algishine creates a more robust surface that better withstands the mechanical and chemical challenges in the oral environment. Future studies could involve long-term immersion tests and mechanical cycling to simulate the wear and tear experienced in the oral cavity.

Although this study offers valuable insights, it has several limitations and suggests areas for future research. This study was conducted under in vitro conditions, which may not fully replicate the complex oral environment. Future studies should include in vivo evaluations to confirm these findings under actual clinical conditions. While coffee, tea, and red wine are common staining agents, future studies should explore a broader range of substances, including different dietary habits and oral hygiene products, to provide a more comprehensive understanding of color stability.

## Conclusions

This study highlights the potential of Algishine, a novel polishing agent derived from pulverized old alginate impressions, in significantly enhancing the color stability of PMMA interim restorations compared to traditional pumice. The use of Algishine not only improves the esthetic longevity of interim restorations but also promotes sustainable practices in dental offices. By offering a smoother, less porous surface, Algishine reduces the adherence of staining agents, thereby maintaining the visual integrity of PMMA restorations. Further research is warranted to explore long-term effects, broader staining scenarios, and clinical outcomes to fully establish Algishine's role in modern dental practice.
